# Editorial: Unraveling breast cancer complexity: insights from single-cell sequencing and spatial transcriptomics

**DOI:** 10.3389/fimmu.2026.1816191

**Published:** 2026-03-16

**Authors:** Apriliana E. R. Kartikasari, Julie Decock

**Affiliations:** 1 School of Health and Biomedical Sciences, Royal Melbourne Institute of Technology (RMIT) University, Melbourne, VIC, Australia; 2 Translational Oncology Research Center, Qatar Biomedical Research Institute (QBRI), Hamad Bin Khalifa University (HBKU), Qatar Foundation, Doha, Qatar; 3 College of Health and Life Sciences (CHLS), Hamad Bin Khalifa University (HBKU), Qatar Foundation, Doha, Qatar

**Keywords:** breast cancer, cancer immune regulation, drug resistance, metastasis, single-cell RNA (scRNA) sequencing, spatial transcriptomic, tumor heterogeneity

Breast cancer research is at a pivotal crossroads, where traditional bulk analyses are no longer sufficient to address the most pressing clinical questions. Two powerful technologies—single-cell sequencing and spatial transcriptomics—provide unprecedented opportunities to reveal the hidden architecture of breast tumors, improve our understanding of breast cancer biology and transform how we diagnose and treat this disease. This Research Topic focuses precisely on these advances with the goal to generate meaningful impact on real-world patient outcomes.

Several studies in this Research Topic used single-cell sequencing and spatial transcriptomics to gain insights into genomic regulation, tumor maintenance mechanisms, and cancer stemness in breast cancer. The study by Miao et al. integrated single-cell sequencing, spatial transcriptomics, and bulk transcriptomics to identify a high synthetic-lethal malignant cell subtype in triple-negative breast cancer (TNBC), characterized by stem-like features. Through machine learning approaches and experimental validation, they established Kinesin Family Member 22 (KIF22) and Kirsten Rat Sarcoma Viral Oncogene Homolog (KRAS) as a synthetic lethal gene pair in TNBC, highlighting a promising therapeutic strategy for synthetic lethality-based drug development. Moreover, Li et al. used chromatin regulator-based molecular subtyping derived from single-cell data to identify three prognostically distinct breast cancer groups. They experimentally validated the chromatin remodeler Achaete-Scute Family BHLH Transcription Factor 1 (ASCL1) as an epigenetic mediator of chemotherapy resistance, suggesting potential clinical relevance of chromatin state. Additionally, integrated transcriptomic and single-cell analyses by Zhen et al. identified the stem cell-related and telomere maintenance genes Jun Proto-Oncogene (JUN), Nuclear Factor Kappa B Subunit 1 (NF-κB1), and Specificity Protein 1 (SP1) as potential biomarkers linked to extracellular matrix remodeling and oxidative stress. These findings provide additional evidence of the increasingly recognized role of the extracellular matrix, reviewed by Li et al. (1), as an active regulator of metastasis and recurrence, rather than merely serving as a structural support.

Another central theme emerging in this Research Topic is immune regulation within the tumor microenvironment. A study by Wu et al. used integrative multi-omics approaches and comprehensively validated Early Growth Response 3 (EGR3) as a dual-function regulator of tumor suppression and immunomodulation in immune-cold breast cancer subtypes. Increased EGR3 expression was associated with enhanced infiltration of CD8+ effector T cells, positioning EGR3 as both a biomarker and a potential therapeutic target. Zhen et al. further reported increased expression of immune checkpoint molecules in breast cancer tissues enriched in activated B and Natural Killer (NK) cells compared to normal tissues. Moreover, an integrative analysis by Wang et al. (2) using single-cell sequencing and bulk transcriptomic data demonstrated that tumor-associated macrophages (TAMs) are strongly correlated with clinical outcomes in triple-negative breast cancer. They identified the expression of Carboxypeptidase-vitellogenic-like (CPVL) and Macrophage Scavenger Receptor 1 (MSR1) in TAMs as potential key determinants of patient prognosis. Similarly, Hou et al. performed integrative analyses of single-cell sequencing and bulk transcriptomic data to develop a prognostic NK cell–related gene signature capable of predicting survival, immunotherapy response, and chemotherapeutic sensitivity. Killer cell lectin-like receptor subfamily B member 1 (KLRB1) and cyclin D2 (CCND2) were validated as key NK-related prognostic biomarkers in this study. Together, these studies emphasize the importance of studying immune cell heterogeneity in breast tumors given their prognostic and therapeutic value.

The Research Topic also includes comprehensive reviews that synthesize current knowledge and outline future directions in the field. Hawsawi et al. highlight recent advancements in single-cell sequencing technologies and analytical approaches. The integration of single-cell sequencing with multi-omics frameworks to decode translational implications and improve clinical interpretation is further detailed by Han et al.An et al. discusses the evolution of spatial transcriptomics technologies and outline their capacities to resolve tumor heterogeneity and guide individualized therapy. Additionally, in this Research Topic, Wang et al. developed a tandem mass spectrometry platform that enables simultaneous quantification of multiple steroid hormones, revealing relationships between local hormone levels and tumor characteristics.

Integrating single-cell and spatial data has the potential to refine patient stratification for targeted therapies, identify new vulnerabilities for drug development, and reveal early molecular indicators of treatment resistance. This is particularly relevant to improve our current understanding of drug resistance and metastasis—long-standing challenges that continue to claim lives. By mapping the molecular underpinnings of resistant clones and metastatic niches, single-cell sequencing and spatial transcriptomics can pave the way for the development of truly individualized treatment strategies.
